# A real-world pharmacovigilance study of amivantamab-related cardiovascular adverse events based on the FDA adverse event reporting system (FAERS) database

**DOI:** 10.1038/s41598-024-55829-5

**Published:** 2024-04-25

**Authors:** Rui Sun, Zhen Ning, Henan Qin, Wenhe Zhang, Yibin Teng, Chenxing Jin, Jiwei Liu, Aman Wang

**Affiliations:** 1 Department of Oncology, The First Affiliated Hospital of Dalian Medical University, Dalian, China; 2 Department of General Surgery, The First Affiliated Hospital of Dalian Medical University, Dalian, China

**Keywords:** Cancer, Cancer epidemiology, Cancer prevention, Cancer screening, Oncology, Cancer, Cancer epidemiology, Cancer prevention, Cancer screening, Cancer therapy

## Abstract

Amivantamab is the first dual-specificity antibody targeting EGFR and MET, which is approved for the treatment of locally advanced or metastatic non-small cell lung cancer (NSCLC) with EGFR exon 20 insertion mutations. Cardiovascular toxicities related to amivantamab have not been reported in the CHRYSALIS study. However, the occurrence of cardiovascular events in the real world is unknown. To comprehensively investigate the clinical characteristics, onset times, and outcomes of cardiovascular toxicities associated with amivantamab. The Food and Drug Administration Adverse Event Reporting System (FAERS) database from 1st quarter of 2019 to the 2nd quarter of 2023 was retrospectively queried to extract reports of cardiovascular adverse events (AEs) associated with amivantamab. To perform disproportionality analysis, the reporting odds ratios (RORs) and information components (ICs) were calculated with statistical shrinkage trans-formation formulas and a lower limit of the 95% confidence interval (CI) for ROR (ROR025) > 1 or IC (IC025) > 0 with at least 3 reports was considered statistically significant. A total of 20,270,918 eligible records were identified, among which 98 records were related to cardiovascular events associated with amivantamab. 4 categories of cardiovascular events exhibited positive signals: venous thrombotic diseases, abnormal blood pressure, arrhythmia, and pericardial effusion. Venous thrombotic diseases and abnormal blood pressure were the two most common signals. The median time to onset (TTO) for cardiovascular AEs was 33 days. The cumulative incidence within 90 days was 100% for cardiac failure, 75% for stroke, 63.16% for arrhythmia, 50% for sudden death, and 44.18% for venous thrombotic diseases. Death accounted for 16.3% of all cardiovascular AEs associated with amivantamab. The mortality rates for Major Adverse Cardiovascular Events (MACE) were up to 60%. This pharmacovigilance study systematically explored the cardiovascular adverse events of amivantamab and provided new safety signals based on past safety information. Early and intensified monitoring is crucial, and attention should be directed towards high-risk signals.

## Introduction

Amivantamab is a fully human bispecific antibody targeting epidermal growth factor receptor (EGFR) and mesenchymal-epithelial transition factor (MET) to inhibit ligand-dependent receptor activation, promote downregulation of cell surface receptors, and induce Fc-dependent trogocytosis and antibody-dependent cellular cytotoxicity^1^. Based on the results of a multicenter, nonrandomized, open-label, multicohort clinical trial (CHRYSALIS, NCT 02609776), the FDA granted accelerated approval for amivantamab on May 21, 2021, for the treatment of patients with locally advanced or metastatic non-small cell lung cancer (NSCLC) with EGFR exon 20 insertion (ex20ins) mutations, whose disease has progressed on or after platinum-based chemotherapy^2^. The overall response rate (ORR) was 40% with median duration of response (DOR) of 11.1 months^2^. Amivantamab was the first bispecific antibody approved for solid tumors, it entered a market offering several existing anti-EGFR mAb therapies.

As a novel targeted agent, amivantamab brings definite clinical benefits to NSCLC patients but also inevitably leads to toxicity. In the CHRYSALIS study, common (≥ 20%) adverse reactions included rash, infusion-related reactions, paronychia, musculoskeletal pain, dyspnea, nausea, edema, cough, fatigue, stomatitis, constipation, vomiting, and pruritus^2^. Cardiovascular toxic events related to amivantamab have not been reported in this trial. However, patients enrolled in clinical trials are rigorously selected and do not represent the occurrence of cardiovascular events in the real world. As the clinical use of amivantamab expands, it is essential to investigate its cardiovascular toxicity based on post-market data and time to onset (TTO) to ensure the safety of patients.

This pharmacovigilance study based on the Adverse Event Reporting System Database of the US Food and Drug Administration (FAERS) was conducted to detect the potential cardiovascular toxicities of amivantamab.

## Results

### Baseline characteristics

**Figure 1:**
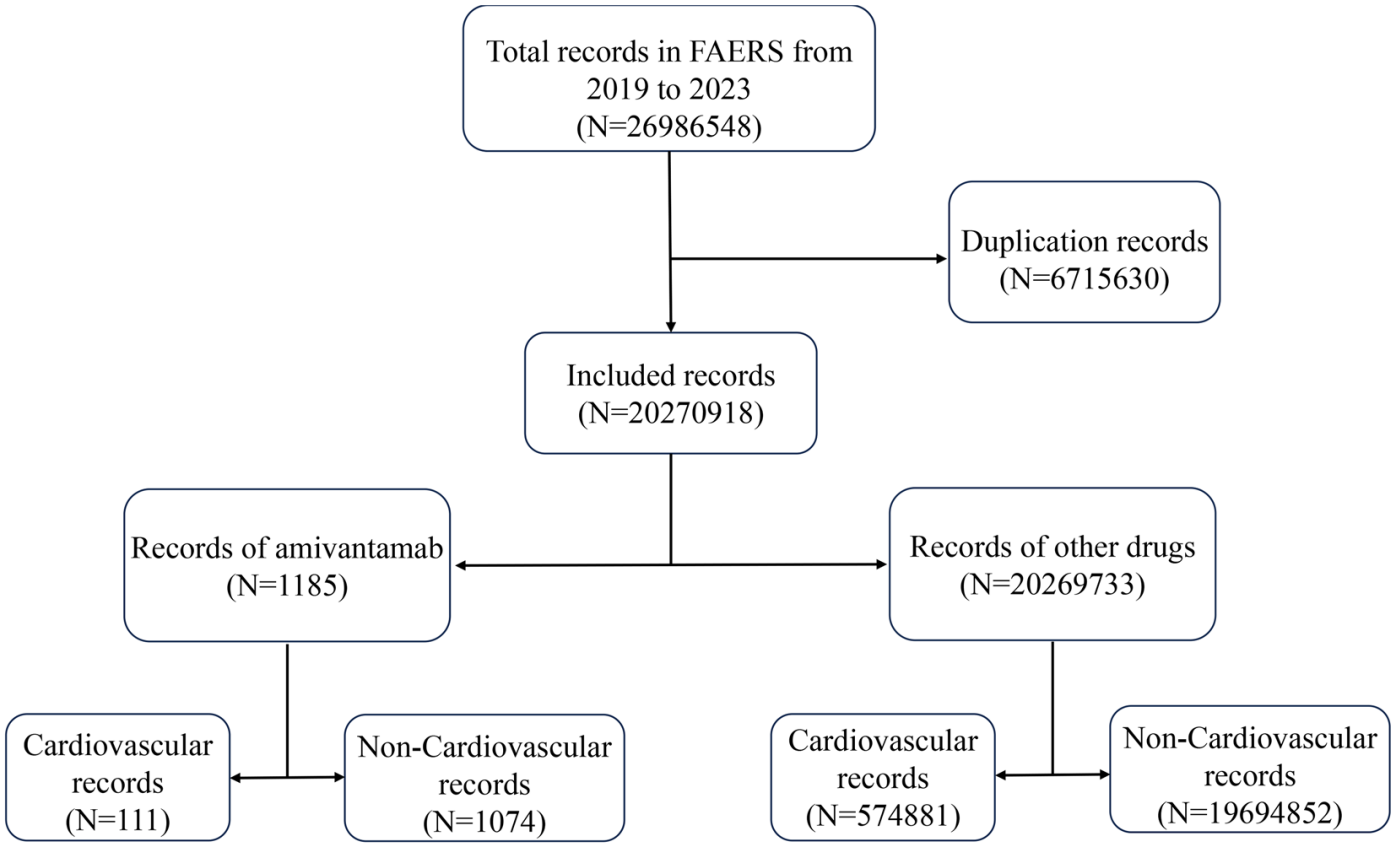
Study flow diagram for included cases identified from quarterly files in the US Food and Drug Administration Adverse Event Reporting System (FAERS) from the first quarter of 2019 to the second quarter of 2023.

Among the 20,270,918 reports recorded in FAERS in the study period from the first quarter of 2019 to the second quarter of 2023, a total of 1185 AEs reports were related to amivantamab. After exclusion of duplicate cases (590 reports), 595 reports were included in the present analysis, with 98 cases (16.47%) being related to cardiovascular AEs (Fig. 1). Table 1 shows the characteristics of AEs reports associated with amivantamab in FAERS. We analyzed cardiovascular and non-cardiovascular AEs related to amivantamab, as well as demographic differences between Major Adverse Cardiovascular Events (MACE) and non-MACE cases. Out of 497 cases, 98 experienced some form of cardiovascular AEs, including 88 non-MACE cases and 10 with MACE. The median age of patients with cardiovascular AEs was 66 years, while patients with MACE were slightly older (69 years). Cardiovascular AEs with amivantamab were chiefly submitted by health professionals (98.0%) and mainly from Asia (41.8%) and North America (33.7%). Cardiovascular AEs was associated with more severe clinical outcomes than non-cardiovascular toxicity (86.7% vs. 64.4%, p < 0.001) and resulted in higher rates of hospitalization (61.2% vs. 34.6%, p = 0.005) and life-threatening (16.3% vs. 6.8%, p = 0.041) conditions.

**Table 1:** Baseline characteristics of cardiovascular toxicity records associated with amivantamab in FAERS from 2019–2023.

	Non cardiovascular toxicity (N = 497)	Any cardiovascular toxicity (N = 98)	*P*	Non-MACE (N = 88)	MACE (N = 10)	*P*
Sex						
Female (%)	206 (41.45)	58 (59.18)	0.238	54 (61.36)	4 (40.00)	0.152
Male (%)	143 (28.77)	30 (30.61)		25 (28.41)	5 (50.00)	
Unknown (%)	148 (29.78)	10 (10.20)		9 (10.23)	1 (10.00)	
Age						
Median (Q1, Q3)*	64 (56, 71)	66 (56, 71)		65 (56, 70.5)	69 (60, 73.5)	
18–45 (%)	13 (2.62)	3 (3.06)	0.743	2 (2.27)	1 (10.00)	0.234
45–65 (%)	114 (22.94)	32 (32.65)		31 (35.23)	1 (10.00)	
65–75 (%)	76 (15.29)	28 (28.57)		24 (27.27)	4 (40.00)	
≥ 75 (%)	36 (7.24)	13 (13.27)		11 (12.50)	2 (20.00)	
Unknown (%)	258 (51.91)	22 (22.45)		20 (22.73)	2 (20.00)	
Type of reporter						
Doctor (%)	273 (54.93)	70 (71.43)	0.005	61 (69.32)	9 (90.00)	0.384
Pharmacist (%)	185 (37.22)	26 (26.53)		25 (28.41)	1 (10.00)	
Consumer (%)	39 (7.85)	2 (2.04)		2 (2.27)	0 (0)	
Year of report						
2021 (%)	74 (14.89)	8 (8.16)	0.100	7 (7.95)	1 (10.00)	0.404
2022 (%)	222 (44.67)	41 (41.84)		35 (39.77)	6 (60.00)	
2023 (%)	201 (40.44)	49 (50.00)		46 (52.27)	3 (30.00)	
State of the reporting country						
North America (%)	291 (58.55)	33 (33.67)	< 0.001	31 (35.23)	2 (20.00)	0.271
Asia (%)	120 (24.14)	41 (41.84)		34 (38.64)	7 (70.00)	
Europe (%)	67 (13.48)	16 (16.33)		15 (17.05)	1 (10.00)	
South America (%)	17 (3.42)	8 (8.16)		8 (9.09)	0 (0)	
Oceania (%)	2 (0.40)	0 (0)		0 (0)	0 (0)	
Serious outcome						
Serious (%)	320 (64.39)	85 (86.73)	< 0.001	75 (85.23)	10 (100.0)	0.350
Non-serious (%)	177 (35.61)	13 (13.27)		13 (14.77)	0 (0)	
Death						
Yes (%)	50 (10.06)	16 (16.33)	0.478	10 (11.36)	6 (60.00)	0.002
No (%)	270 (54.33)	69 (70.40)		65 (73.86)	4 (40.00)	
Unknown (%)	177 (35.61)	13 (13.27)		13 (14.77)	0 (0)	
Hospitalization—initial or prolonged						
Yes (%)	172 (34.61)	60 (61.22)	0.005	55 (62.50)	5 (50.00)	0.128
No (%)	148 (29.78)	25 (25.51)		20 (22.73)	5 (50.00)	
Unknown (%)	177 (35.61)	13 (13.27)		13 (14.77)	0 (0)	
Life-threatening						
Yes (%)	34 (6.84)	16 (16.33)	0.041	10 (11.36)	6 (60.00)	0.002
No (%)	286 (57.55)	69 (70.40)		65 (73.86)	4 (40.00)	
Unknown (%)	177 (35.61)	13 (13.27)		13 (14.77)	0 (0)	

*Quarter 1–3 (Q1–Q3).

Among all cardiovascular AEs, MACE resulted in a higher proportion of life-threatening (60.0% vs. 11.4%, p = 0.002) and fatal events (60.0% vs. 11.4%, p = 0.002) compared to non-MACE cardiovascular AEs.

### Disproportionality analysis

**Figure 2:**
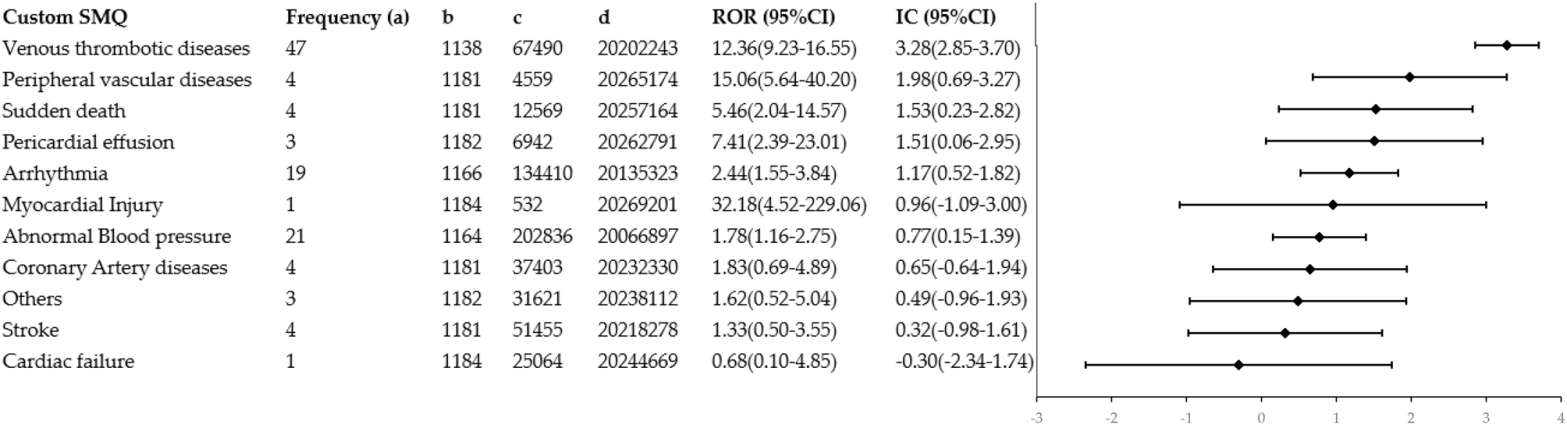
Cardiovascular events in Custom SMQ with disproportionality analysis in the FAERS database (see supplementary materials S1 for PT affiliations). a, frequency of targeted adverse events in the target drug population (amivantamab-cardiovascular AEs); b, total adverse events (a + b) minus a value occurred in the target drug population; c, target total number of adverse events (a + c) minus a value; d, total adverse events in the background population (n)-a-b-c.

We screened for cardiovascular adverse events (AEs) Preferred Terms (PTs) and categorized them into customized cardiovascular AEs Standardized Medical Dictionary for Regulatory Activities Queriers (SMQs) (see supplementary materials S1 for PT affiliations). Figure 2 summarizes the results of the disproportionality analyses. Overall, among the 11 custom cardiovascular AEs SMQs, the top three cardiovascular AEs with the highest number of reported cases were venous thrombotic diseases (N = 47, ROR: 12.36, 95% CI: 9.23–16.55, IC: 3.28, 95% CI: 2.85–3.70), abnormal blood pressure (N = 21, ROR: 1.78, 95% CI: 1.16–2.75, IC: 0.77, 95% CI: 0.15–1.39), and arrhythmia (N = 19, ROR: 2.44, 95% CI: 1.55–3.84, IC: 1.17, 95% CI: 0.52–1.82). Among the 11 SMQs, 4 SMQs showed signals: venous thrombotic diseases, abnormal blood pressure, arrhythmia, and pericardial effusion.

**Figure 3:**
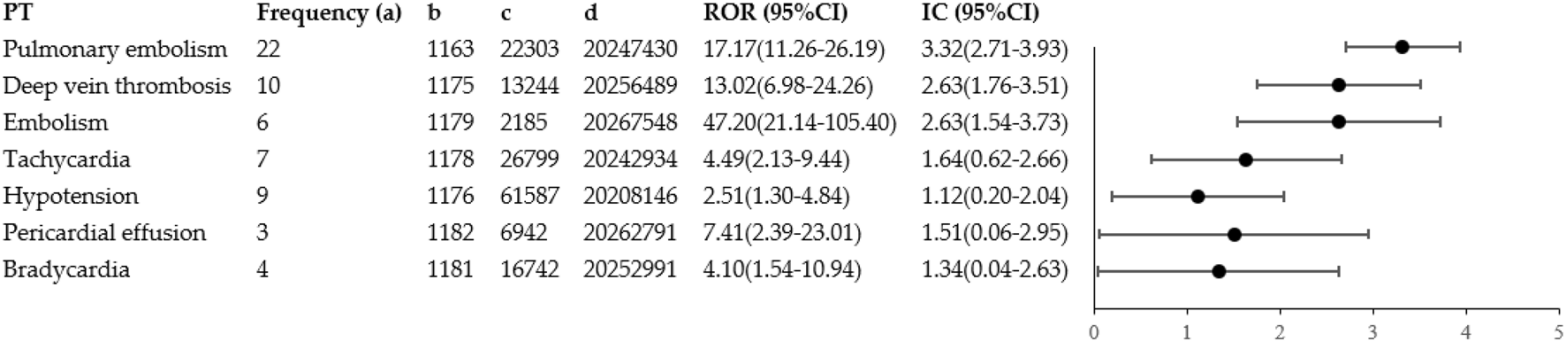
The signals in preferred terms (PTs) associated with cardiovascular toxicities (see supplementary materials S1 for PT affiliations). a, Frequency of targeted adverse events in the target drug population (amivantamab-cardiovascular AEs); b, total adverse events (a + b) minus a value occurred in the target drug population; c, target total number of adverse events (a + c) minus a value; d, total adverse events in the background population (n)-a-b-c.

Notably, among the aforementioned 4 SMQs, 7 PTs are of significance. (Fig. 3). As for the signal strength, pulmonary embolism (N = 22, ROR: 17.17, 95% CI: 11.26–26.19) was the most common cardiovascular toxicity and demonstrated the strongest signal value. The next strongest signal was for deep vein thrombosis (N = 10, ROR: 13.02, 95% CI: 6.98–24.26), followed by hypotension (N = 9, ROR: 2.51, 95% CI: 1.30–4.84).

### TTO

**Figure 4:**
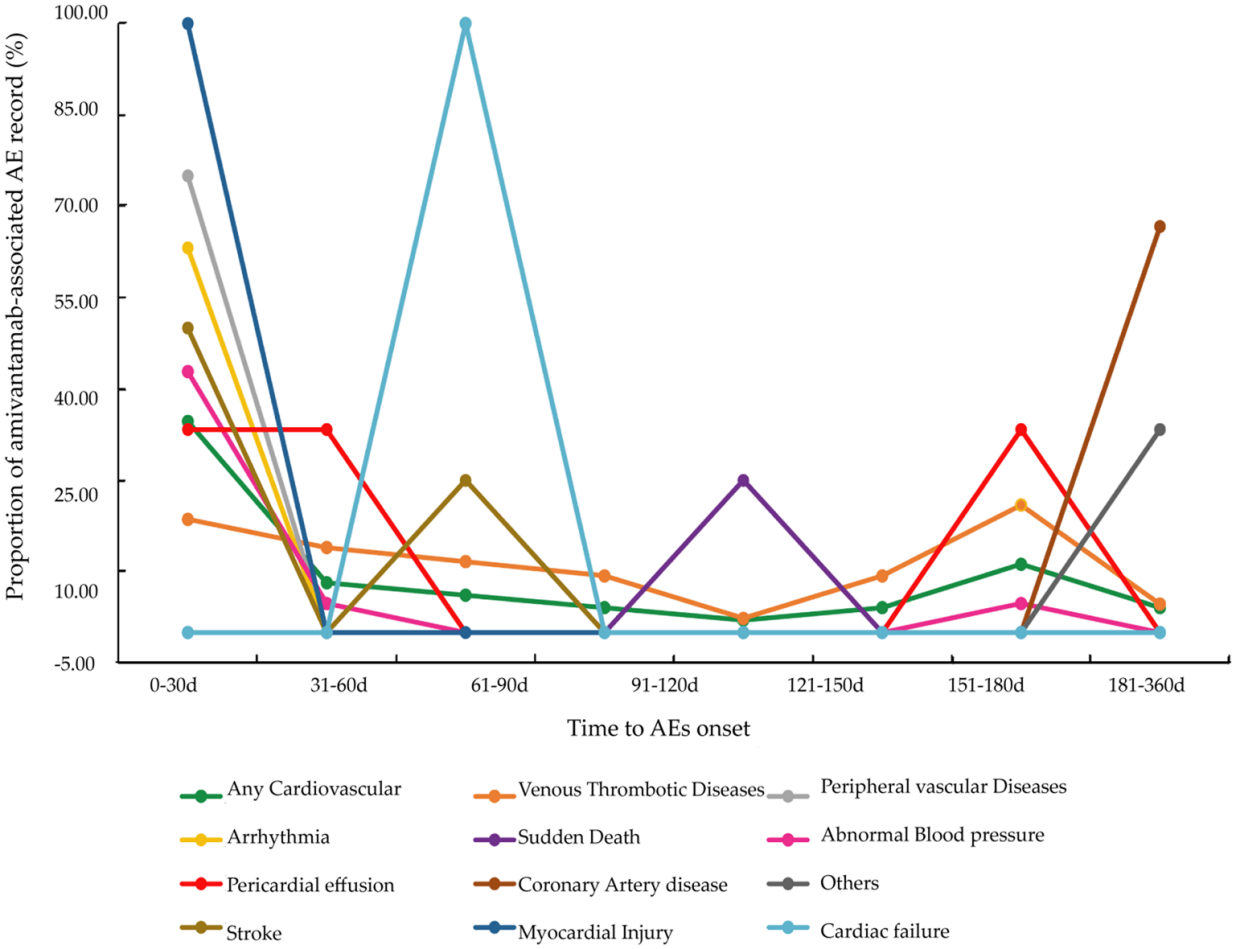
Time to AEs onset for cardiovascular toxicities.

The Fig. 4 illustrates the differences in TTO for the aforementioned 11 custom SMQs related to cardiovascular AEs. Overall, the median TTO for cardiovascular AEs was 33 days, and Q1–Q3 was 2–151 days.

Arrhythmia had the shortest TTO of 0–13 days. The median TTO of sudden death was 23 days (Q1–Q3: 12–133 days), for heart failure 77 days, and for venous thrombophilia 94 days (Q1–Q3: 33–216 days). Coronary artery diseases displayed the longest median TTO of 570 days (Q1–Q3: 563–577 days). Furthermore, all myocardial injury occurred within the initial month of administration. The cumulative incidence within 90 days was 100% for cardiac failure, 75% for stroke, 63.16% for arrhythmia, 50% for sudden death, and 44.18% for venous thrombotic diseases.

### Outcome

**Figure 5:**
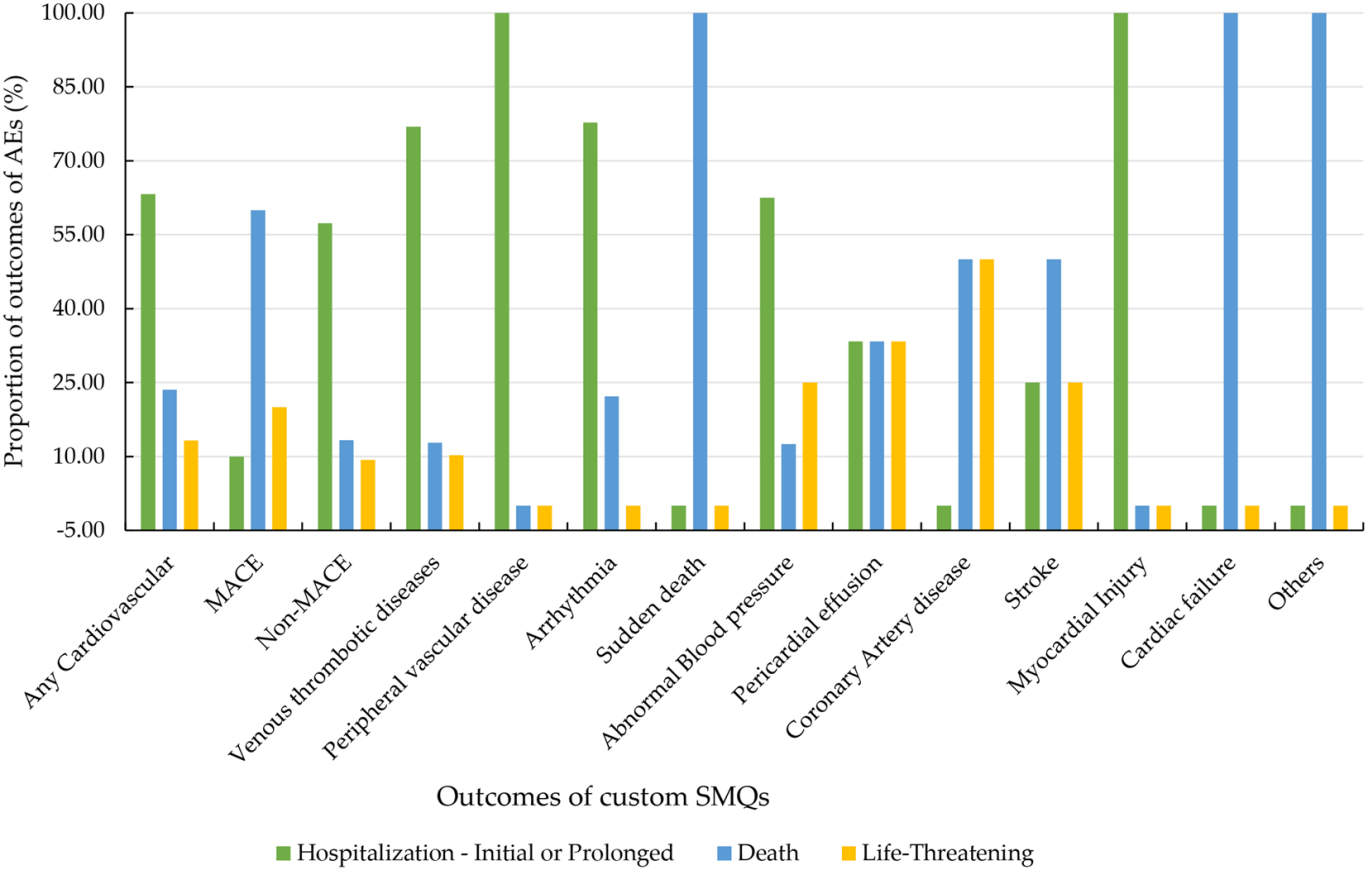
Outcomes of cardiovascular toxicities.

We further explored the outcomes associated with these 11 cardiovascular AEs. Of note, death accounted for 16.3% of all cardiovascular AEs associated with amivantamab. Figure 5 showed the higher risk level outcomes including death, life-threatening events, and caused or prolonged hospitalization proportions according to specific SMQs with significant signals.

Notably, the mortality rates for MACE were 60%. The mortality rates for sudden death and heart failure were both 100%. The mortality rate was 50% for coronary heart disease and stroke and 33.3% for pericardial effusion. The reporters concluded that all death cases reported in the FAERS database were related to adverse reactions of the targeted drug, however, this inevitably introduces some bias. The probability of life-threatening coronary heart disease was 50%, 33.3% for pericardial effusion, and 25% for abnormal blood pressure and stroke. Myocardial injury and peripheral vascular disease caused or prolonged hospitalization in 100% of cases, arrhythmia in 77.8%, and venous thrombosis disease in 76.9%, but these AEs were rarely life-threatening.

## Discussion

Amivantamab, the first approved EGFR and MET bispecific antibody for treating locally advanced or metastatic NSCLC with EGFR exon 20 insertion (ex20ins) mutations, has garnered attention for its promising clinical prospects in recent years^2^. However, as a novel targeted drug, its toxicity, especially cardiovascular safety, cannot be ignored. To date, no large prospective randomized or retrospective studies have reported the cardiovascular toxicity of amivantamab. However, in the real world, most lung cancer patients are elderly with multiple co-morbidities, so the cardiovascular safety of amivantamab in the real world deserves further investigation. To the best of our knowledge, this is the first comprehensive pharmacovigilance study on cardiovascular toxicity associated with amivantamab by leveraging the FAERS database, involving the frequency, spectrum, timing, and outcomes of cardiovascular toxicity.

Amivantamab exerts antitumor effects by simultaneously targeting the EGFR and MET signaling pathways^1^. The specific mechanisms of amivantamab-related cardiovascular toxicity remain unclear, but we can draw insights from the mechanisms of cardiovascular toxicity of other targeted drugs (e.g., anti-Her-2 targeted agents), which may be related to the inhibition of specific targets^3–5^. The EGFR and MET pathways play important roles in the development of the cardiovascular system, maintenance of normal cardiovascular function and cardiovascular stress^6,7^. Blockade of these targets may disrupt key cardiovascular signaling pathways, which may lead to cardiovascular toxicity.

In our study, a total of 595 reports were included, with 98 cases experiencing varying degrees of cardiovascular adverse events, of which 88 were non-MACE and 10 were MACE. Amivantamab-related cardiovascular adverse events were predominantly reported by health professionals (98%), with the majority originating from Asia (41.8%) and North America (33.7%). In comparison to non-cardiovascular toxicity, cardiovascular adverse events were associated with more severe clinical outcomes, leading to higher hospitalization rates, and life-threatening situations. The proportion of life-threatening events and fatal events caused by MACE was higher than non-MACE cardiovascular adverse events.

Our results identified higher than expected reports for 4 SMQs, including venous thrombotic diseases, abnormal blood pressure, arrhythmia, and pericardial effusion. Venous thrombotic diseases, particularly pulmonary embolism, was the most common cardiovascular toxicity observed. Previous studies have demonstrated that patients with advanced lung cancer are at a heightened risk of developing venous thromboembolism (VTE), and the administration of specific anti-tumor therapy further escalates the incidence^8–10^. The role of EGFR inhibition in directly inducing endothelial damage or increasing thrombus formation has not been conclusively established. In vitro-cultured human microvascular endothelial cells show enhanced expression of plasminogen activator upon EGF stimulation, but it seems more plausible that anti-EGFR monoclonal antibodies may prolong the exposure of endothelial structures induced by combination therapy, favoring platelet activation, leukocyte adhesion, oxidative stress, coagulation, and inflammation-all factors contributing to thromboembolism^11^. The correlation between MET inhibition and thrombosis and the underlying mechanisms are unknown. MET inhibitors (e.g., capmatinib, etc.) have been reported to cause VTE events in NSCLC patients^12^. Infusion reactions were one of the most common toxicities in phaseICHRYSALIS trial of amivantamab, occurring in more than 60% of cases^2^. Hypotension, one of the clinical manifestations of infusion reactions, showed a high incidence in our study. The primary PT in arrhythmia is tachycardia, with several case reports pointing to EGFR-TKI-associated ventricular tachycardia^13–15^. The potential pathophysiological mechanisms of arrhythmia are still unclear. One hypothesis is that EGFR-TKIs may alter the myocardial sarcoplasmic reticulum calcium homeostasis and induce repolarization abnormalities^16,17^. The mechanism of pericardial effusion has not been fully elucidated, but previous studies suggested that the non-target effects of EGFR-TKI on the immune system may contribute to serositis^18^. EGFR-TKI-induced serositis may occur at any time, ranging from 3 weeks to 2 years from the initial treatment^19^, and is considered to be dose-dependent^20^. Although pleural effusion is the most common presentation, concomitant pericardial effusion may occur in up to 29% of cases^18^.

As for the TTO of cardiovascular AEs, no studies have reported the disparity of timing according to different types of cardiovascular AEs associated with amivantamab. Our present study compared the TTO of specific cardiovascular AEs based on SMQs, which offered valuable insights. The median TTO for cardiovascular AEs was 33 days, with all myocardial injuries occurring within the first month of drug administration. Within the initial 3 months, the cumulative incidence rates for cardiac failure, stroke, arrhythmia, sudden death, and venous thrombotic diseases were 100%, 75%, 63.16%, 50%, and 44.18%, respectively. This suggests a need for heightened attention to these cardiovascular adverse events during amivantamab treatment, especially in the first 3 months. Identifying differences in the onset times among different cardiovascular AEs in clinical practice may guide distinct monitoring strategies.

Regarding the outcomes of cardiovascular adverse events, this study indicates that death accounts for 16.3% of all cardiovascular AEs related to amivantamab, which is higher than other drugs. MACE has a mortality rate of up to 60% and a worse outcome. The mortality rates for sudden death and cardiac failure are both 100%. The mortality rate was 50% for coronary heart disease and stroke and 33.3% for pericardial effusion. The probability of life-threatening coronary heart disease was 50%, 33.3% for pericardial effusion, and 25% for abnormal blood pressure and stroke. Myocardial injury and peripheral vascular disease result in higher rates of caused or prolonged hospitalization, but are rarely life-threatening.

The cardiovascular toxicity of the dual-targeted antibody amivantamab appears to be greater than that of TKIs. Therefore, safety is also a critical consideration when establishing clinical protocols. This may suggest the need for enhanced cardiovascular assessment and monitoring when opting for dual-targeted therapy. This study carries limitations that merit acknowledgment. First, the FAERS database is reliant on voluntary data submissions. Consequently, inherent biases such as under-reporting, over-reporting, and data incompleteness are unavoidable and unquantifiable. Second, the causal relationship between AEs and medication cannot be precisely confirmed in retrospective studies. Third, the majority of reporting data in the FAERS database originates from North America (particularly the United States), European countries, and Japan, which may introduce regional bias into the results. More prospective studies and longer follow-ups are still required to validate our findings.

## Methods

### Study design and database

This observational pharmacovigilance study utilized the FAERS database, covering the period from 1st quarter of 2019 to the 2nd quarter of 2023. The FAERS database, maintained by the FDA, is an open repository that aggregates adverse event reports from various sources, including patients, healthcare professionals, and drug manufacturers^21^. It is one of the most common used databases for mining signals of adverse drug reactions in pharmacovigilance.

### Data cleaning

Before data analysis, reports with the same gender, age, reporting country, adverse event, drug name, start, and end dates were defined as duplicate data and subjected to duplicate data removal. This study did not employ missing data imputation methods, as FAERS is a spontaneous database with a significant proportion of missing data for its variables.

Both generic and brand names were used to identify the target drug, Amivantamab. AEs were encoded in conjunction with the PT as per the Medical Dictionary for Regulatory Activities (MedDRA). Several PTs were grouped into SMQ and System Organ Class (SOC) to describe medical conditions or systemic diseases.

In this study, we customized classifications and utilized 11 SMQs related to cardiovascular events as adverse events and PTs related to the cardiovascular disease SOC to detect cardiovascular toxicity associated with amivantamab (see supplementary materials S1 for PT affiliations).

### Statistical analysis

Disproportionality analysis, also known as case/non-case analysis (cardiovascular AEs/ non-cardiovascular AEs), was employed in this study, and it is one of the most common signal detection methods in pharmacovigilance^22^. Two commonly used methods for disproportionality analysis are the ROR and IC^23,24^. In this study, both methods were utilized for signal detection. The detailed calculation process of ROR, and IC can be found in supplementary materials S2.

A signal is indicated when IC025 exceeds 0 or when ROR025 exceeds 1, with an expected count of more than 3. All analyses were conducted using SAS version 9.4 (SAS Institute, Inc., Cary, NC, USA).

Different PTs may describe very similar adverse events, which are considered separate adverse events. This can potentially impact the accuracy of results. To address this, we customized categorization based on the SMQ, as detailed in the supplementary materials S1. We further categorized patients into groups based on the following criteria: cardiovascular events, non-cardiovascular events, MACE, non-MACE, cardiovascular serious reports (outcomes including life-threatening, hospitalization—initial or prolonged, disability, death, and congenital anomaly), and non- serious reports. We conducted an analysis of demographic characteristics among these different groups, employing statistical methods such as the Chi-squared test.

### Supplementary Information

Supplementary Information 1.

Supplementary Information 2.

Supplementary Information 3.

## Data Availability

All data generated or analyzed during this study are included in the supplementary material S3. The datasets presented in this study can be found in online repositories. The names of the repository/repositories and accession number(s) can be found in the following: https://fis.fda.gov/extensions/FPD-QDE-FAERS/FPD-QDE-FAERS.html.
