# Supplementary material for: A real-world pharmacovigilance study of amivantamab-related cardiovascular adverse events based on the FDA adverse event reporting system (FAERS) database
Source: Sci Rep. 2024 Apr 25;14:9552. doi: 10.1038/s41598-024-55829-5 (PMC11045761; doi:10.1038/s41598-024-55829-5)
Supplement: Supplementary file 1 — Supplementary Information 1. [file 41598_2024_55829_MOESM1_ESM.docx]

Classification of Cardiovascular Toxicity

The underlined term represents Major Adverse Cardiovascular Events (MACE).

Abnormal Blood pressure：

Hypotension：Hypotension

Blood Pressure Decreased

Orthostatic Hypotension

Hypertension：Hypertension

Blood Pressure Increased

Arrhythmia：

Tachycardia：Tachycardia

Heart Rate Increased

Bradycardia：Bradycardia

Heart Rate Decreased

Atrial Fibrillation：Atrial Fibrillation

Arrhythmia：Arrhythmia

Ventricular Fibrillation：Ventricular Fibrillation

Coronary Artery disease：

Myocardial Infarction：Myocardial Infarction

Myocardial Rupture：Myocardial Rupture

Coronary Artery Disease：Coronary Artery Disease

Acute Coronary Syndrome：Acute Coronary Syndrome

Arteriosclerosis Coronary Artery：Arteriosclerosis Coronary Artery

Myocardial Injury：Troponin I Increased

Cardiomyopathy：Stress Cardiomyopathy

Cardiac Failure：Cardiac Failure

Pericardial Effusion：Pericardial Effusion

Venous thrombotic diseases

Pulmonary Embolism：Pulmonary Embolism

Pulmonary Infarction

Deep Vein Thrombosis：Deep Vein Thrombosis

Embolism Venous：Embolism Venous

Venous Thrombosis

Jugular Vein Thrombosis

Superior Sagittal Sinus Thrombosis：Superior Sagittal Sinus Thrombosis

Embolism

Thrombosis

Peripheral Embolism

Venous thrombosis limb

Peripheral vascular disease：

Vasculitis

Vascular Access Complication

Vascular Pain

Vascular Rupture

Sudden death：Sudden Death

Sudden Cardiac Death

Cardio-Respiratory Arrest

Stroke：

Cerebrovascular Accident：Cerebrovascular Accident

Cerebral Infarction：Cerebral Infarction

Cerebral Thrombosis

Ischaemic Stroke：Ischaemic Stroke

Others：Cardiac Disorder

Cardiovascular Disorder

Cardiovascular Insufficiency
