# Supplementary material for: A real-world pharmacovigilance study of amivantamab-related cardiovascular adverse events based on the FDA adverse event reporting system (FAERS) database
Source: Sci Rep. 2024 Apr 25;14:9552. doi: 10.1038/s41598-024-55829-5 (PMC11045761; doi:10.1038/s41598-024-55829-5)
Supplement: Supplementary file 2 — Supplementary Information 2. [file 41598_2024_55829_MOESM2_ESM.docx]

## ROR

| **Method** | **Computational formula** | **Threshold value** |
| --- | --- | --- |
| ROR | $ROR=\frac{(a/c)}{(b/d)}=\frac{ad}{bc}$  $SE(lnROR)=\sqrt{(\frac{1}{a}+\frac{1}{b}+\frac{1}{c}+\frac{1}{d})}$  $95\%CI=e^{ln(ROR)\pm1.96\sqrt{(\frac{1}{a}+\frac{1}{b}+\frac{1}{c}+\frac{1}{d})}}$ | A signal is indicated when ROR025 exceeds 1 and a≥3 |

| **Items** | **Target adverse event reports** | **Other adverse event reports** | **Total** |
| --- | --- | --- | --- |
| Target drug | a | b | a+b |
| Other drugs | c | d | c+d |
| Total | a+c | b+d | n=a+b+c+d |

1)a, frequency of targeted adverse events in the target drug population;

2)b, total adverse events (a+b) minus a value occurred in the target drug population;

3)c, target total number of adverse events (a+c) minus a value;

4)d, total adverse events in the background population (n)-a-b-c.

## BCPNN

| **Method** | **Computational formula** | **Threshold value** |
| --- | --- | --- |
| BCPNN | IC=${log}_{2}\frac{p(x,y)}{p(x)p(y)}={log}_{2}\frac{a(a+b+c+d)}{(a+b)(a+c)}$  E(IC)=${log}_{2}\frac{(a+\gamma11)(a+b+c+d+\alpha)(a+b+c+d+\beta)}{（a+b+c+d+\gamma）(a+b+\alpha1)(a+c+\beta1)}$  V(IC)=$\frac{1}{{(ln2)}^{2}}\{\left[ \frac{\left( a+b+c+d \right)-a+\gamma-\gamma11}{\left( a+\gamma11 \right)\left( 1+a+b+c+d+\gamma\right)} \right]+\left[ \frac{\left( a+b+c+d \right)-\left( a+b \right)+\alpha-\alpha1}{\left( a+b+\alpha1 \right)\left( 1+a+b+c+d+\alpha\right)} \right]+\left[ \frac{\left( a+b+c+d \right)-\left( a+c \right)+\beta-\beta1}{\left( a+c+\beta1 \right)\left( 1+a+b+c+d+\beta\right)} \right]\}$  $\gamma=\gamma11\frac{(a+b+c+d+\alpha)(a+b+c+d+\beta)}{(a+b+\alpha1)(a+c+\beta1)}$  *IC-2SD=E(IC)-2*$\sqrt{V(IC)}$  $\alpha1=\beta1=1；\alpha=\beta=2；\gamma11=1$ | A signal is indicated when IC025 exceeds 0 and a≥3 |

| **Items** | **Target adverse event reports** | **Other adverse event reports** | **Total** |
| --- | --- | --- | --- |
| Target drug | a | b | a+b |
| Other drugs | c | d | c+d |
| Total | a+c | b+d | n=a+b+c+d |

1)a, frequency of targeted adverse events in the target drug population;

2)b, total adverse events (a+b) minus a value occurred in the target drug population;

3)c, target total number of adverse events (a+c) minus a value;

4)d, total adverse events in the background population (n)-a-b-c.
